# Zone 1 total arch replacement with frozen elephant trunk for type A dissection: early-to-mid-term outcomes

**DOI:** 10.1186/s13019-026-04324-1

**Published:** 2026-06-03

**Authors:** Yusuke Takei, Ikuko Shibasaki, Masahiro Tezuka, Takashi Kato, Takeshi Ogasawara, Takayuki Hori, Toshiyuki Kuwata, Go Tsuchiya, Masashi Kawamura, Hirotsugu Fukuda

**Affiliations:** 1 Department of Cardiac and Vascular Surgery, Dokkyo Medical University School of Medicine, 880 Kitakobayashi, Mibu-machi, Shimotsuga-gun, Tochigi, 321-0293 Japan; 2 Department of Cardiovascular Surgery, Maebashi Red Cross Hospital, Maebashi, Gunma Japan; 3 Department of Cardiovascular Surgery, Iwaki City Medical Center, Iwaki, Fukushima Japan; 4 Department of Fundamental Education Premedical Sciences, Dokkyo Medical University, Mibu-machi, Shimotsuga-gun, Tochigi, Japan

**Keywords:** Stanford type A aortic dissection, Frozen elephant trunk, Left subclavian artery, Distal stent graft-induced new entry

## Abstract

**Background:**

Total arch replacement with frozen elephant trunk is widely used to treat acute Stanford type A aortic dissection. Zone 1 proximalization, which requires concomitant extra-anatomical left subclavian artery bypass, may provide a practical balance between safety and technical complexity. Although the early outcomes of this procedure have previously been reported, comprehensive mid-term data, particularly regarding left subclavian artery bypass durability, remain limited. This study aimed to evaluate the early and mid-term outcomes of zone 1 proximalization in total arch replacement with frozen elephant trunk for acute Stanford type A aortic dissection.

**Methods:**

This retrospective observational study included 80 patients with acute type A aortic dissection who underwent zone 1 total arch replacement with frozen elephant trunk, which requires concomitant extra-anatomical left subclavian artery bypass, between 2014 and 2025 at two institutions. Primary outcomes were postoperative mortality and freedom from aorta- and left subclavian artery bypass-related events. Secondary outcomes included perioperative complications and reinterventions. Event-free survival was assessed using Kaplan–Meier analysis.

**Results:**

Thirty-day and in-hospital mortality rates were 8.8% and 10.0%, respectively. Stroke occurred in 12.5% of patients and spinal cord injury in 5.0%. Freedom from aorta-related events was 88.4%, 84.0%, and 81.5% at one, three, and five years, respectively; corresponding rates for left subclavian artery bypass-related events were 95.6%, 91.5%, and 86.4%, respectively. Of the 21 aorta-related events, 62% involved the downstream aorta and were managed with endovascular repair. The eight left subclavian artery bypass-related events were mostly asymptomatic occlusions caused by technical issues. Distal stent graft-induced new entry, a major late complication, was associated with a larger zone 4 diameter.

**Conclusions:**

This descriptive study provided early and mid-term outcome data for zone 1 total arch replacement with frozen elephant trunk in patients with acute type A aortic dissection. The long-term durability of the left subclavian artery bypass remains a concern. Considering the anatomical features of the patient and ensuring vigilant follow-up may reduce downstream events, making this procedure a viable option for selected patients.

**Supplementary Information:**

The online version contains supplementary material available at 10.1186/s13019-026-04324-1.

## Background

Total arch replacement with frozen elephant trunk (TARFET) is widely used to treat acute Stanford type A aortic dissection (ATAAD), as it promotes favorable aortic remodeling and reduces downstream reintervention rates compared to conventional total arch replacement or the classical elephant trunk technique [1–4]. From a technical perspective, proximalization of the distal anastomosis to Ishimaru zones 0–2 minimizes the extent of arch dissection and facilitates secure anastomosis compared with zone 3 anastomosis [5–9]. Zone 0 offers the greatest degree of proximalization but complicates reoperation owing to the proximity of arch branches; zone 2 provides only limited proximalization. Zone 1, which requires extra-anatomical left subclavian artery (LSA) bypass yet allows secure exposure and reliable distal anastomosis, represents a practical balance between safety and technical complexity [10]. However, LSA bypass is typically routed through a narrow intercostal space to reach the axillary artery, forming a long and tortuous course that is prone to kinking or occlusion, potentially compromising graft patency [11]. The length of the device also influences outcomes: shorter devices increase the risk of distal stent graft-induced new entry (d-SINE) due to the spring-back force, whereas longer ones raise the risk of spinal cord injury (SCI) [12–15].

Although the early outcomes of zone 1 TARFET have been reported previously [16], comprehensive mid-term data, particularly regarding d-SINE incidence and LSA bypass durability, remain limited. Therefore, this study aimed to evaluate the early- and mid-term outcomes of zone 1 TARFET, with a focus on patient survival and freedom from aorta- and LSA bypass-related events.

## Methods

### Study population

This retrospective observational study was conducted at two institutions: Dokkyo Medical University Hospital and Maebashi Red Cross Hospital. Data from patients who underwent surgery for ATAAD between December 2014 and February 2025 at Dokkyo Medical University Hospital and between April 2018 and February 2025 at Maebashi Red Cross Hospital were reviewed. ATAAD was diagnosed based on clinical presentation and contrast-enhanced computed tomography (CT), with acuteness defined as presentation within 14 days of symptom onset. Patients with ATAAD who underwent zone 1 TARFET were included in the study. TARFET is generally indicated in patients aged ≤ 70 years with a patent false lumen (FL) and those aged > 70 years with an entry tear distal to the ascending aorta or an arch dilatation ≥ 40 mm. The exclusion criteria were as follows: performance of hemiarch replacement, conventional total arch replacement, fenestrated TARFET [17], TARFET in other zones, or DeBakey type II dissection or intramural hematoma without arch dilatation; presence of severe comorbidities; frailty, as represented by a Clinical Frailty Scale score of ≥ 6 [18]; and poor ventricular function, as represented by an ejection fraction < 20%. Comorbidities included hypertension, cerebrovascular disease, coronary artery disease, chronic kidney disease, and Marfan’s syndrome and were defined according to standard clinical criteria. CT-based analyses were not performed on patients who did not survive to follow-up or on those who underwent intramural hematoma or DeBakey type II dissection with arch dilatation. Clinical, operative, and follow-up data were obtained from the institutional databases. Malperfusion syndrome (MPS) was classified as M1 (coronary), M2 (supra-aortic), or M3 (spinal, visceral, renal, or iliac), in accordance with the TEM (Type of dissection, Entry tear location, Malperfusion) criteria [19].

### Surgical procedure

The technique of zone 1 TARFET was based on the open stent graft method originally described by Kato et al. [11], with institutional modifications. All patients underwent zone 1 TARFET under moderate hypothermic circulatory arrest (25 °C) with selective cerebral perfusion. Cardiopulmonary bypass was established via right axillary and femoral arterial cannulation, with venous drainage from the right atrium. An 8-mm graft was anastomosed to the left axillary artery and routed through the intercostal space into the mediastinum for extra-anatomical LSA bypass. Subsequently, this graft was anastomosed to the dedicated LSA branch of the four-branched graft within the mediastinum. The graft length and course were carefully adjusted to avoid kinking. At 25 °C, lower-body circulatory arrest was initiated, the arch was transected in zone 1, and a J Graft FROZENIX FET (Japan Lifeline Co., Ltd., Tokyo, Japan) was inserted. The FET diameter was determined from preoperative CT images by measuring the total aortic diameter at the anticipated landing site, and a device not exceeding 90% of this diameter was selected. Regarding FET length, a 9-cm device was used as the institutional standard, achieving a balance between adequate distal coverage and spinal cord injury risk; longer devices (12–15 cm) were used in selective cases with a concomitant distal arch aneurysm. The distal anastomosis between the four-branched graft and distal stump was reinforced with an external felt strip, and the proximal stump was sealed with BioGlue surgical adhesive (Artivion, Kennesaw, GA, USA). Lower-body perfusion was resumed via a side branch, followed by anastomosis to the proximal stump. After cardiac resumption, supra-aortic vessels were reconstructed sequentially: the left common carotid artery, brachiocephalic artery, and left axillary artery graft (see the video in Additional file 1).

### Outcomes

The primary outcomes were postoperative mortality (30-day and in-hospital) and freedom from aorta- and LSA bypass-related events. Aorta-related events included anastomotic pseudoaneurysm, d-SINE, an FL-related aneurysm ≥ 55 mm, rupture, graft infection, and stenosis. Indications for reintervention were as follows: anastomotic pseudoaneurysm was generally treated with open repair, d-SINE and graft stenosis were treated with TEVAR, and FL-related aneurysm reaching the elective repair threshold (≥ 55 mm or rapid expansion) was treated with open repair or TEVAR depending on the anatomical extent. LSA bypass-related events included pseudoaneurysm, graft infection, and occlusion.

The secondary outcomes were perioperative complications and the reintervention rate. Brachial plexopathy was defined as newly developed postoperative upper extremity numbness or fine motor dysfunction, with new cerebral infarction excluded on neuroimaging.

### Imaging protocol and measurement

CT was routinely performed before discharge, six months and one year postoperatively, and annually thereafter. Contrast-enhanced CT was used as standard, with non-contrast CT utilized in patients with renal dysfunction or an allergy. Non-contrast CT enabled diameter and angle measurements, but FL thrombosis could not be assessed. All images were acquired using a 64-detector row SOMATOM Sensation 64 CT scanner (Siemens Healthineers, Erlangen, Germany) and a slice thickness of 0.5–1.0 mm. Images were reconstructed in axial and multiplanar formats for morphological assessment. Diameters were measured co-axially on short-axis images (zones 4 and 5). The FET tip level was recorded relative to vertebral level. FL thrombosis was categorized as patent, partial, complete, or regression and dichotomized as patent or thrombosed. FET and spring-back angles were measured as described by Furutachi et al. [15] (Additional file 2).

### Statistical analysis

Continuous variables were tested for normality using the Shapiro–Wilk test. Group comparisons were performed using the Student’s t-test (with Welch’s correction if variances were unequal), Mann–Whitney U test for continuous variables, and Fisher’s exact test for categorical variables. The time origin for survival analyses was the date of surgery, and the time scale was time since surgery. Event-free survival was estimated using Kaplan–Meier analysis with 95% confidence intervals; curves were truncated when < 10 patients remained at risk. Exploratory subgroup analysis evaluated overall survival stratified by MPS (M1 and/or M2) using the log-rank test and Cox regression adjusted for age and sex. Given the clinical importance of d-SINE, a subgroup analysis was also performed according to its presence or absence, which was restricted to patients who underwent follow-up CT. Morphological parameters, including FET angle, spring-back angle, aortic diameter, and the presence of FL thrombosis, were compared using the above statistical methods. Analyses were performed using EZR software version 1.68 (Saitama Medical Center, Jichi Medical University, Saitama, Japan) [20] based on R software version 4.2.3, and the Python libraries Pandas, NumPy, and SciPy. A two-sided p value < 0.05 was considered statistically significant. Missing data were handled by complete-case analysis and occurred only in CT-based variables such as FL thrombosis in non-contrast CT and FET tip level in early mortality cases; no clinical or operative data were missing. As a retrospective cohort, the study size was determined by all consecutive eligible cases during the accrual period; no a priori power calculation was performed. No sensitivity analyses were prespecified or performed due to the single-arm design of the study and its limited sample size.

## Results

### Patient demographics

**Fig. 1:**
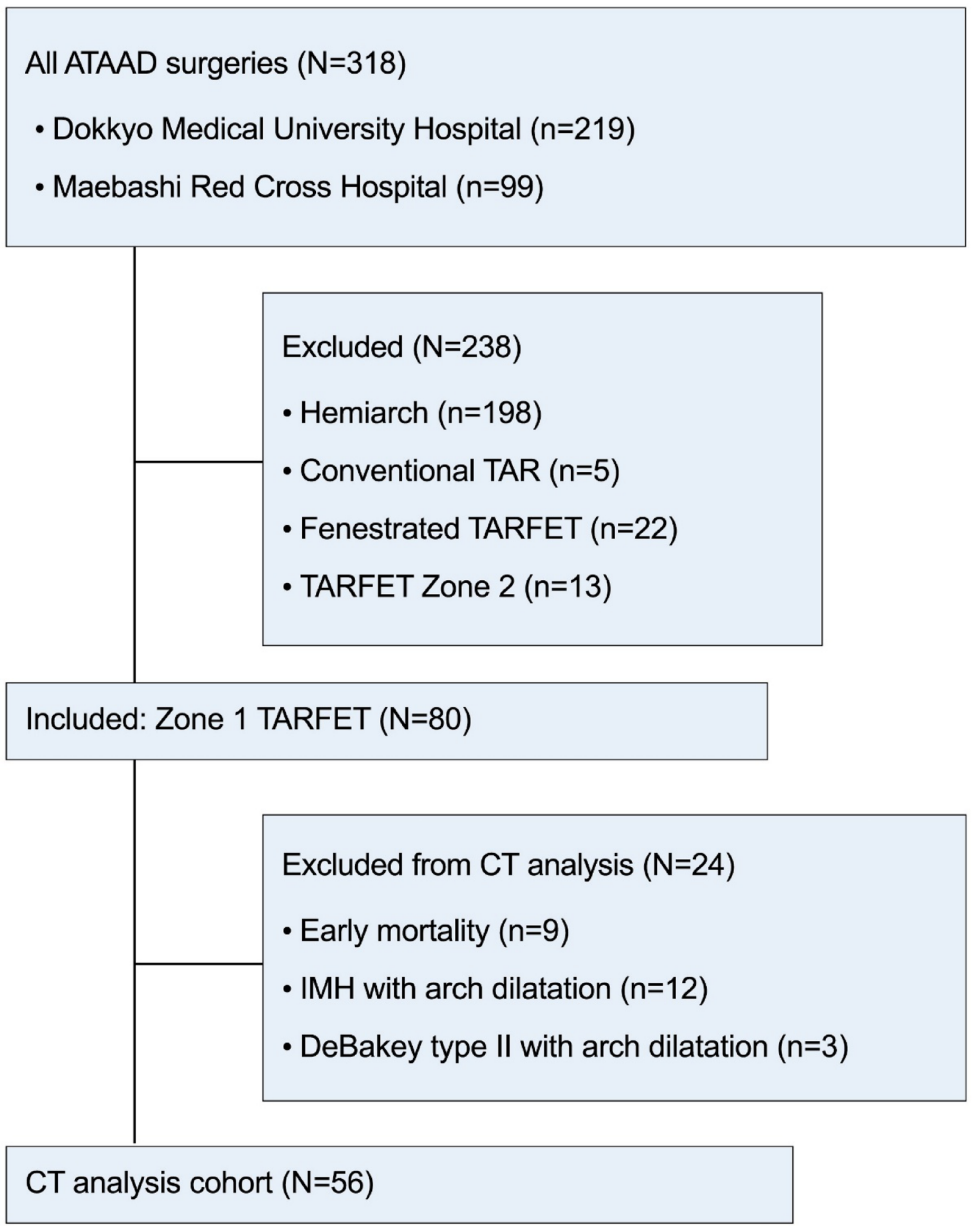
Flow diagram of the study. ATAAD, acute Stanford type A aortic dissection; CT, computed tomography; IMH, intramural hematoma; TAR, total arch replacement; TARFET, total arch replacement with frozen elephant trunk

A total of 80 patients underwent zone 1 TARFET at Dokkyo Medical University Hospital and Maebashi Red Cross Hospital during the study period, with a median follow-up of 3.8 (interquartile range, 1.0–6.2) years. The patient selection process is presented in Fig. 1, and the preoperative patient characteristics are summarized in Table 1. None of the patients had undergone a prior sternotomy. MPS was observed in 21 patients and was classified as M1 in 5 patients (including 1 who required preoperative coronary intervention), M2 in 7 patients (4 who exhibited neurologic deficits as a result of bilateral carotid dissection and 3 who had carotid occlusion or dissection but were asymptomatic), and M3 in 12 patients (lower limb ischemia in 10, preoperative paraplegia in 1, and renal dysfunction in 1). No intestinal malperfusion was observed. Three patients had a combined malperfusion involving multiple territories.

**Table 1:** Patient characteristics

Characteristic	*N* = 80
Age, years	59.6 ± 12.4
Body mass index, kg/m^2^	24.2 [21.5, 26.4]
Body surface area, m^2^	1.76 ± 0.22
Male	58 (72.5)
Hypertension	56 (70.0)
Cerebral vascular disease	3 (3.8)
Coronary artery disease	2 (2.5)
Chronic kidney disease	10 (12.5)
Marfan’s syndrome	1 (1.3)
DeBakey type II with arch dilatation	3 (3.8)
Intramural hematoma with arch dilatation	12 (15.0)
Cardiac tamponade	5 (6.3)
Malperfusion syndrome	21 (26.3)
M1, n, +/-	5, 5/0
M2, n, +/-	7, 4/3
M3, n, +/-	12, 8/4

Values are presented as mean ± standard deviation, median [interquartile range], or n (%). +/-, with or without clinical symptoms. M1, coronary malperfusion; M2, supra-aortic malperfusion; M3, spinal/visceral/renal/iliac malperfusion

### Early outcomes

**Fig. 2:**
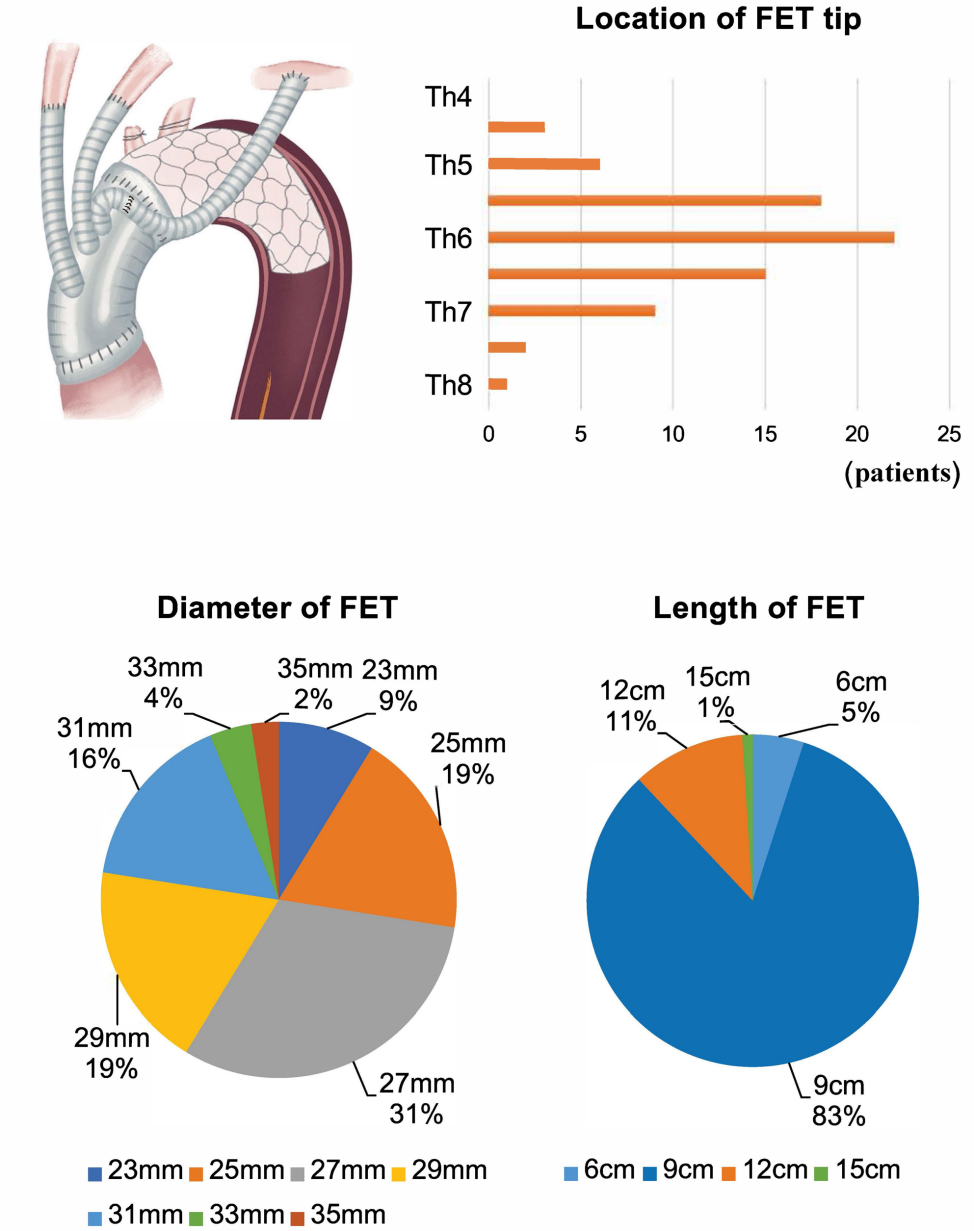
Tip location, diameter, and length of FET. FET, frozen elephant trunk

Operative and perioperative data are presented in Table 2. Most patients received a 9-cm FET, with the tip most often positioned at Th6 (Fig. 2), indicating relatively shallow deployment. The in-hospital mortality rate was 10% (*n* = 8), and the 30-day mortality rate was 8.8% (*n* = 7). Stroke was the leading cause of death (*n* = 5), followed by low cardiac output syndrome (*n* = 2) and mediastinitis (*n* = 1). Two stroke-related deaths occurred in patients with preoperative M2 malperfusion, suggesting that neurological injury may have preceded surgery. Of the two deaths from low cardiac output syndrome, one patient had preoperative M1 malperfusion and the other had a recent history of coronavirus disease 2019. Exploratory subgroup analysis showed poorer survival in the MPS group (hazard ratio 4.86, 95% confidence interval 1.34–17.61, *p* = 0.016) than in the non-MPS group (Additional files 3 and 4).

**Table 2:** Perioperative data

Parameter	*N* = 80
Cardiopulmonary bypass time, min	291 [264, 351]
Lower body circulatory arrest time, min	62 [46, 78]
Selective cerebral perfusion time, min	199 [170, 224]
Concomitant procedures†	13 (16.3)
Bentall, n	6
VSRR, n	4
CABG, n	3
Femoro-femoral bypass, n	1
30-day mortality	7 (8.8)
In-hospital mortality	8 (10.0)
Re-exploration for bleeding	4 (5.0)
Stroke	10 (12.5)
mRS score ≥ 3	7 (8.8)
Spinal cord injury	4 (5.0)
Paraparesis	1 (1.3)
Paraplegia	3 (3.8)
Brachial plexopathy	3 (3.8)

†One patient underwent two concomitant procedures. Values are presented as median [interquartile range] or n (%). CABG, coronary artery bypass grafting; mRS, modified Rankin Scale; VSRR, valve-sparing root replacement

Stroke occurred in 10 patients (12.5%), of whom 5 died, 2 experienced disabling sequelae, and 3 recovered fully. SCI was experienced by four patients (5.0%), one preoperatively and three postoperatively. Details of each SCI case, including lower body circulatory arrest time and FET distal tip location, are provided in Additional file 5. All three patients with postoperative SCI underwent cerebrospinal fluid drainage; one patient fully recovered, while the other two had residual motor deficits. LSA bypass-related brachial plexopathy occurred in three patients (3.8%).

### Mid-term outcomes

**Fig. 3:**
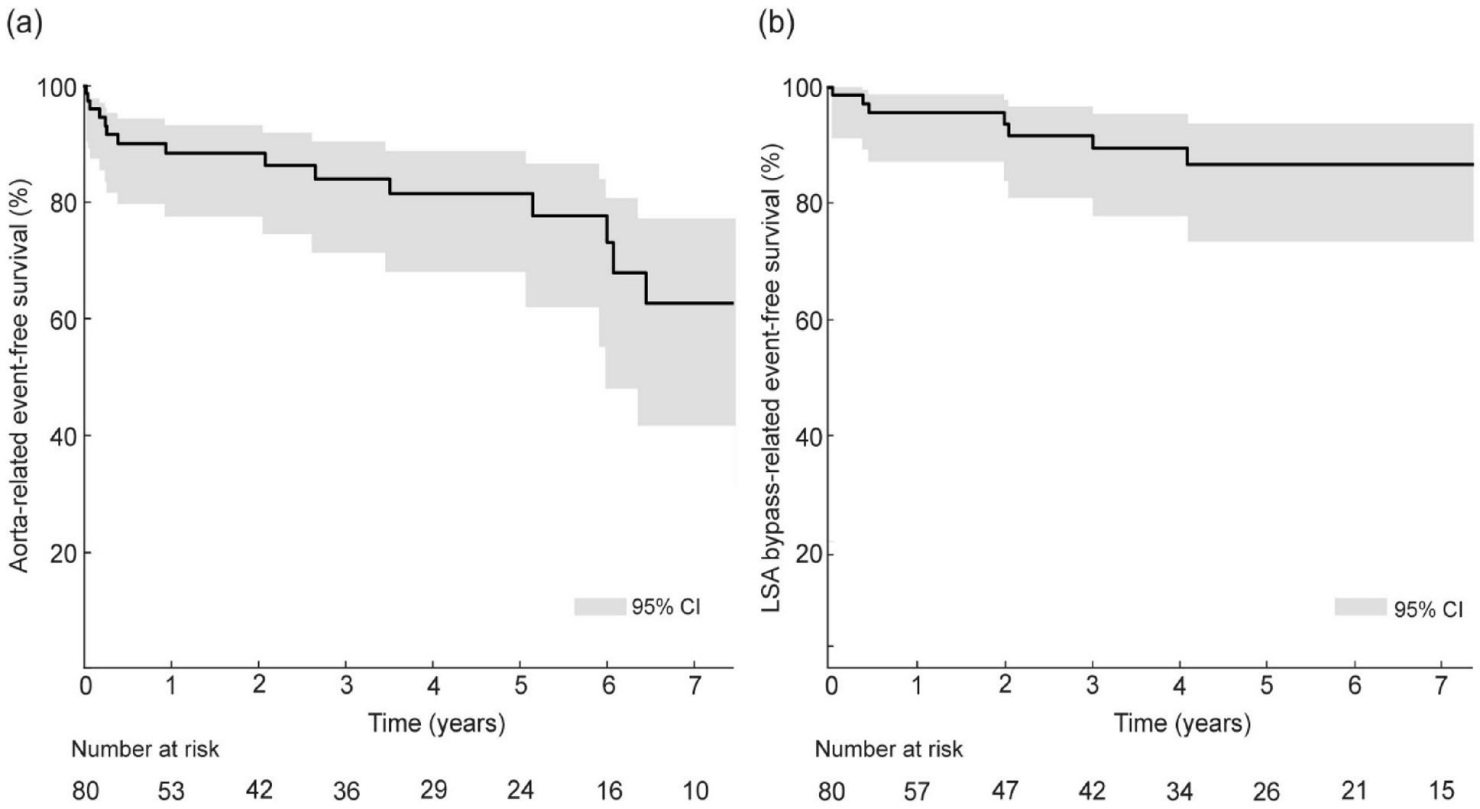
Kaplan–Meier curves showing freedom from (**a**) aorta-related events and (**b**) LSA bypass-related events. CI, confidence interval; LSA, left subclavian artery

At one, three, and five years postoperatively, freedom from aorta-related events was 88.4%, 84.0%, and 81.5%, respectively (Fig. 3a), and freedom from LSA bypass-related events was 95.6%, 91.5%, and 86.4%, respectively (Fig. 3b). A total of 29 events occurred (21 aorta-related and 8 LSA bypass-related) (Table 3). Of these, 20 required reinterventions, the majority with endovascular procedures.

**Table 3:** Aorta- and LSA-related events and reinterventions

Event	Number of events	Reintervention required	
		Open repair	Endovascular
Aorta-related	21	9	9
Pseudoaneurysm^†^	6	5	0
d-SINE	6	0	6
Rupture^‡^	2	0	1
FL expansion ≥ 55 mm^§^	5	3	1
Graft stenosis	1	0	1
Hemolysis	1	1	0
LSA bypass-related	8	0	2
Bypass occlusion	6	0	0
Pseudoaneurysm	2	0	2
Total	29	9	11

†One pseudoaneurysm was small and localized and was managed conservatively. ‡One patient with rupture did not survive to receive treatment. §One patient with FL expansion is currently under surveillance with repair planned

Values are presented as n. d-SINE, distal stent graft-induced new entry; FL, false lumen; LSA, left subclavian artery

**Fig. 4:**
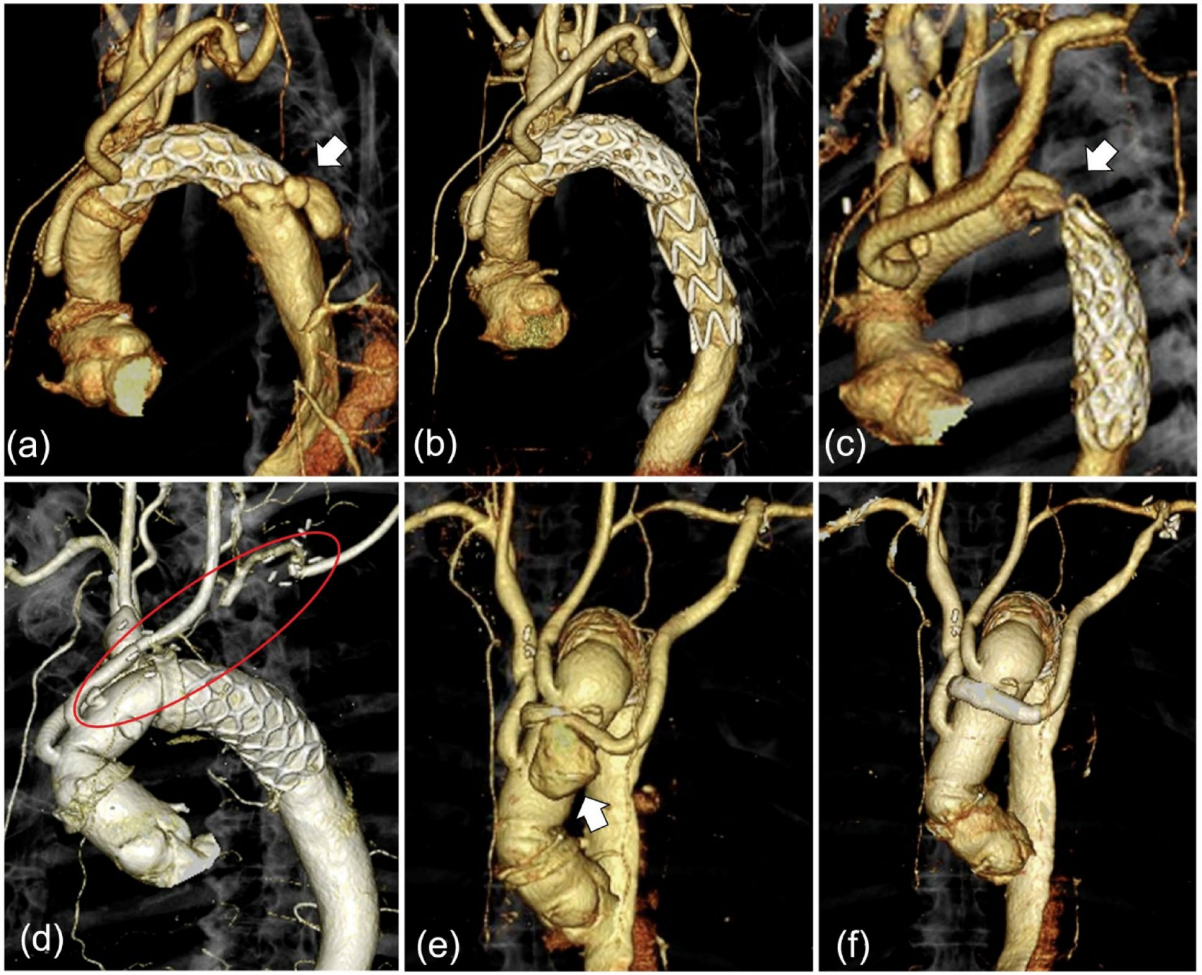
CT findings of aorta- and LSA bypass-related events. (**a**) Distal stent graft-induced new entry (arrow) and (**b**) its exclusion after TEVAR. (**c**) Stenosis at the non-stented frozen elephant trunk segment (arrow), which was subsequently treated with TEVAR. (**d**) LSA bypass graft occlusion (circle). (**e**) Pseudoaneurysm at the LSA anastomosis site (arrow) and (**f**) its exclusion with a peripheral stent graft. CT, computed tomography; LSA, left subclavian artery; TEVAR, thoracic endovascular aortic repair

The most frequently occurring aorta-related events were proximal anastomotic pseudoaneurysm (treated with redo ascending aortic replacement) and d-SINE (Fig. 4a) (treated with thoracic endovascular aortic repair [TEVAR]) (Fig. 4b). FL expansion caused two ruptures, one of which was operable; of the five patients who reached the elective threshold, three underwent open repair, one underwent TEVAR, and one is currently under surveillance. Rare events included graft kinking in the non-stented FET portion (Fig. 4c), which was treated with TEVAR, and hemolysis from the kinking of the four-branched graft, which was treated by segmental graft replacement. Excluding pseudoaneurysms, graft stenosis, and hemolysis, 13 of the 21 aorta-related events (62%; 16% overall) were downstream events. Reintervention was required for 18 of the 21 aorta-related events (86%).

LSA bypass-related events comprised six graft occlusions, all of which were asymptomatic or mildly symptomatic and were managed conservatively (Fig. 4d, Additional file 6), and two anastomotic pseudoaneurysms (Fig. 4e), both treated with VIABAHN peripheral stent grafts (W. L. Gore & Associates, Newark, DE, USA) (Fig. 4f).

Subgroup analysis revealed that the zone 4 diameter at the latest follow-up was larger in patients with d-SINE than in those without d-SINE, whereas no differences were observed between the two groups in terms of the FET angle, spring-back angle, zone 5 diameter, or FL thrombosis (Table 4).

**Table 4:** Comparison of imaging parameters according to the presence of d-SINE

Parameter	Total *N* = 56	d-SINE(-) *n* = 50	d-SINE(+) *n* = 6	*p* value
**FET angle,** $\:^\circ\:$				
** Postoperative**	120 [109, 128]	120 [109, 130]	118 [111, 124]	0.83
** Latest follow-up**	134 [125, 145]	134 [125, 145]	133 [133, 146]	0.43
**Spring-back angle**,$\:^\circ\:$				
** Postoperative**	20 [14, 29]	20 [14, 29]	20 [15, 27]	0.96
** Latest follow-up**	38 [23, 45]	38 [23, 44]	38 [27, 50]	0.35
**Zone 4 aortic diameter**,** mm**				
** Postoperative**	33.7 [31.4, 36.8]	33.6 [31.4, 36.1]	36.5 [33.8, 39.0]	0.17
** Latest follow-up**	33.0 [29.5, 35.4]	32.6 [28.9, 34.9]	44.1 [37.9, 44.7]	0.03
**Zone 5 aortic diameter**,** mm**				
** Postoperative**	31.8 [29.6, 33.8]	31.8 [29.6, 33.5]	32.4 [28.3, 34.5]	1.00
** Latest follow-up**	31.8 [28.2, 36.8]	31.1 [28.1, 36.1]	36.1 [34.3, 41.1]	0.12
**FL thrombosis at zone 4**,** n**				
** Postoperative**	29/56 (51.8)	28/50 (56.0)	1/6 (16.7)	0.10
** Latest follow-up**	37/47 (78.7)	34/42 (81.0)	3/5 (60.0)	0.29
**FL thrombosis at zone 5**,** n**				
** Postoperative**	14/56 (25.0)	14/50 (28.0)	0/6 (0.0)	0.32
** Latest follow-up**	25/47 (53.2)	23/42 (54.8)	2/5 (40.0)	0.65

Continuous data are shown as median [interquartile range] and categorical data as n (%). Numbers may not total due to missing CT data. d-SINE, distal stent graft–induced new entry; FET, frozen elephant trunk; FL, false lumen

## Discussion

This study demonstrated worse survival in patients with preoperative MPS than in patients without MPS. Analysis of aorta- and LSA bypass-related events showed that LSA bypass-related events were generally mild and preventable, whereas most aorta-related events required reintervention; distal aortic enlargement emerged as a key predictor of d-SINE.

The early outcomes reported in the present study are comparable to those of the Japanese national database, which reports the results of TARFET for ATAAD irrespective of the implantation zone (30-day mortality, 9.2–10.4%; stroke, 11.5–14.6%; and SCI, 5.8–6.5%) [1, 21], and those reported by international meta-analyses (mortality, 9.2%; stroke, 9.3%; and SCI, 2.4%) [13]. Specialist high-volume centers have reported more favorable outcomes (30-day mortality, 0.8–2.8%; stroke, 3.7–10.0%; and SCI, 0–3.5%) [4, 10, 22]; however, our results remain within the expected range.

Although the SCI rate of 5.0% found in this study is comparable to data from the Japanese national database [1, 21], it warrants individual case analysis. Excluding one case involving a patient with preoperative paraplegia, three new postoperative SCI cases occurred (3.75%). A case-by-case review (Additional file 5) revealed that each case involved distinct contributing factors, including technical complications and anatomically high-risk patterns. Therefore, SCI in our series reflects individual factors rather than FET length per se. The optimal distal anastomotic zone for TARFET remains debatable. The use of zones 0–2 has been shown to reduce circulatory arrest time and technical complexity compared with zone 3, while maintaining favorable outcomes [5–8]. A recent study suggested that superior results are achieved using zone 0 or 1 compared to zone 2, although the technical challenges of LSA reconstruction bring risks of graft occlusion and anastomotic pseudoaneurysm [9]. Fenestrated [17] or branched [23, 24] FET techniques enable in situ LSA reconstruction but are contraindicated when the LSA is dissected or arises distally. In such cases, an extra-anatomical bypass provides a practical solution. Operating in zone 0 remains technically demanding because FET deployment is more complex, and arch branches, which cluster near the ascending aorta, can complicate reoperations. Zone 1 has therefore emerged as a feasible alternative to zone 0, balancing surgical exposure and anastomotic security with the need for extra-anatomical LSA bypass.

Of the eight LSA bypass-related events observed in the present study, six were graft occlusions, of which two occurred early (within six months postoperatively), three occurred mid-term (between two and three years postoperatively), and one occurred late (seven years postoperatively). A retrospective review of the CT images revealed graft kinking and intercostal narrowing in all but the late case, suggesting that most occlusions occurred due to preventable technical issues. Carotid-subclavian bypass via a supraclavicular incision, connecting the left common carotid artery to the LSA, is an alternative approach that avoids intercostal tunneling and may offer superior durability [25]. However, this technique requires familiarity with supraclavicular dissection, which was not consistent across all surgeons in our series. Therefore, we prioritized standardization of the axillary-to-graft technique following the method described by Kato et al. [11] to ensure procedural consistency. All patients were asymptomatic or mildly symptomatic, likely because of vertebrobasilar collaterals. However, vertebral artery hypoplasia, reported in 10–26% of individuals [26], may limit this compensation, and subclavian steal syndrome can lead to ischemic symptoms under conditions of stress [27], underscoring the need for careful patient follow-up, even in the absence of symptoms.

Aorta-related events in our cohort were broadly consistent with previous studies, which have reported reintervention rates of 10–30% for aorta-related events [4, 28–30]. Of the 21 aorta-related events observed in the present study, the majority involved the downstream aorta, were attributable to d-SINE or FL expansion, and were largely treatable with endovascular repair [31, 32].

A relatively high incidence of proximal anastomotic pseudoaneurysms was observed in the present study. Although case reports have linked surgical adhesives such as BioGlue to late pseudoaneurysm or necrosis [33, 34], a larger series found this risk to be low (0.6%) when adhesives were used appropriately [35]. Incomplete resection of the dissected tissue may also contribute to the incidence of proximal anastomotic pseudoaneurysms. Expert consensus recommends performing a proximal anastomosis at or near the sinotubular junction in healthy tissue [36]. Our findings support this principle, emphasizing the importance of meticulous stump preparation.

Among downstream complications, d-SINE is a major late event, with a reported incidence of 10–20% [15, 37]. In the present study, d-SINE accounted for a substantial proportion of events and was successfully managed with TEVAR. Our subgroup analysis showed that patients with d-SINE had a significantly larger zone 4 diameter at the latest follow-up than that of patients without d-SINE, whereas the FET angle, spring-back angle, and FL thrombosis did not differ significantly between the two groups. These findings align with recent imaging-based studies that identified distal aortic size and progressive expansion as dominant predictors, with FET angulation less relevant [38]. Distal enlargement reflects inadequate aortic remodeling. Longer FETs may promote remodeling and reduce the incidence of d-SINE; however, extension beyond Th8 increases the risk of SCI [39]. Expert consensus, therefore, recommends positioning the FET tip within the straight descending aorta [40] and tailoring the length to each patient rather than uniformly applying a 9-cm device. Nevertheless, in some cases remodeling is not achieved despite an adequate FET depth. In such cases, advanced imaging, such as four-dimensional flow magnetic resonance imaging, can identify abnormal FL flow predictive of negative remodeling [41], allowing early prophylactic TEVAR in high-risk patients.

### Limitations

This study had several limitations. First, it was a retrospective, single-arm study without a comparator group. A comparison with hemiarch replacement or conventional total arch replacement within the same institutional cohort would have been most informative; however, such a contemporaneous comparison was not feasible given that zone 1 TARFET has been our institutional standard throughout the study period. Similarly, comparisons with zone 0, 2, or 3 TARFET were not possible. The involvement of nine surgeons with varying levels of technical expertise, ranging from early-career to senior surgeons, also introduced variability. Second, although relatively large compared with previous studies on zone 1 TARFET, the sample size and event numbers were limited, reducing the statistical power of the subgroup analyses. Third, imaging evaluations were partly constrained by the inability to assess FL thrombosis in non-contrast CT and the lack of CT analysis in patients who died early. In addition, the follow-up period was insufficient to analyze late outcomes beyond 10 years.

## Conclusions

This descriptive study provided early and mid-term outcome data for zone 1 TARFET in patients with ATAAD at two institutions. Technical considerations, particularly the durability of the LSA bypass and the optimal FET length needed to balance remodeling with neurological safety, remain important. Zone 1 TARFET may represent a feasible option for suitable patients; however, comparative studies are needed to establish its role relative to other proximalization strategies. Future studies with larger cohorts and prospective designs are warranted to validate the findings of the present study and further refine patient selection and procedural strategies.

## Supplementary Information

Supplementary Material 1: Zone 1 total arch replacement with the frozen elephant trunk surgical procedure. The video begins after establishing cardiopulmonary bypass with arterial inflow through the right axillary and left femoral arteries and venous drainage from the right atrium and the subsequent initiation of core cooling. An 8-mm graft was anastomosed to the previously exposed left axillary artery and routed into the mediastinum through the intercostal space. Perfusion to the left subclavian artery was provided via the left axillary graft. At a bladder temperature of 25 °C, lower body circulatory arrest was initiated, and the aortic arch was opened. The origins of the LCCA and LSA were ligated. Following the insertion of a selective cerebral perfusion cannula into the distal LCCA, cerebral perfusion was initiated by clamping the brachiocephalic artery; this step was omitted from the video. A frozen elephant trunk was deployed into the descending aorta from zone 1, and the distal arch stump was fashioned with a 4-0 polypropylene running suture reinforced with an external felt strip. Distal anastomosis to a four-branched graft was completed, and lower body perfusion was resumed through the side branch. In the case shown here, the LCCA was reconstructed first, followed by proximal anastomosis to the ascending aorta. The proximal stump was prepared using internal and external felt strips secured with surgical adhesive, and the proximal anastomosis was completed with a 4-0 polypropylene running suture. After resumption of cardiac activity, the brachiocephalic artery was reconstructed, and the LSA branch of the four-branched graft was anastomosed to the left axillary graft previously routed into the mediastinum. The LSA bypass was carefully tailored in terms of length and course to avoid kinking or stenosis. LCCA, left common carotid artery; LSA, left subclavian artery.

Supplementary Material 2

Supplementary Material 3

Supplementary Material 4

Supplementary Material 5

Supplementary Material 6

## Data Availability

The datasets generated and/or analyzed during the current study are available from the corresponding author upon reasonable request.

## Abbreviations

ATAAD: Acute Stanford type A aortic dissection
CT: Computed tomography
d-SINE: Distal stent graft-induced new entry
FET: Frozen elephant trunk
FL: False lumen
LSA: Left subclavian artery
MPS: Malperfusion syndrome
SCI: Spinal cord injury
TARFET: Total arch replacement with frozen elephant trunk
TEVAR: Thoracic endovascular aortic repair

## Declarations

### Meeting presentation

This work was presented at the 55th annual meeting of the Japanese Society of Cardiovascular Surgery in Shimonoseki, Yamaguchi, Japan, on 21 February 2025.

## References

[1] Ogino H, Kumamaru H, Motomura N, Fujiyoshi T, Shimahara Y, Azuma N, et al. Current status of surgical treatment for acute aortic dissection in Japan: nationwide database analysis. J Thorac Cardiovasc Surg. 2025;169:11-23.e1. 38056765 10.1016/j.jtcvs.2023.11.044

[2] Dohle DS, Tsagakis K, Janosi RA, Benedik J, Kühl H, Penkova L, et al. Aortic remodelling in aortic dissection after frozen elephant trunk. Eur J Cardiothorac Surg. 2016;49:111–7. 25715431 10.1093/ejcts/ezv045

[3] Inoue Y, Matsuda H, Omura A, Seike Y, Uehara K, Sasaki H, et al. Comparative study of the frozen elephant trunk and classical elephant trunk techniques to supplement total arch replacement for acute type A aortic dissection. Eur J Cardiothorac Surg. 2019;56:579–86. 31005998 10.1093/ejcts/ezz104

[4] Yoshitake A, Tochii M, Tokunaga C, Hayashi J, Takazawa A, Yamashita K, et al. Early and long-term results of total arch replacement with the frozen elephant trunk technique for acute type A aortic dissection. Eur J Cardiothorac Surg. 2020;58:707–13. 32236552 10.1093/ejcts/ezaa099

[5] Choudhury RY, Basharat K, Zahra SA, Tran T, Rimmer L, Harky A, et al. Proximalization is Advancement - Zone 3 frozen elephant trunk vs Zone 2 frozen elephant trunk: A literature review. Vasc Endovascular Surg. 2021;55:612–8. 33754903 10.1177/15385744211002493

[6] Tsagakis K, Osswald A, Weymann A, Demircioglu A, Schmack B, Wendt D, et al. The frozen elephant trunk technique: impact of proximalization and the four-sites perfusion technique. Eur J Cardiothorac Surg. 2021;61:195–203. 34378023 10.1093/ejcts/ezab295 PMC8759516

[7] Panfilov DS, Kozlov BN, Pryakhin AS, Kopeva KV. Frozen elephant trunk technique with different proximal landing zone for aortic dissection. Interact Cardiovasc Thorac Surg. 2021;33:286–92. 33846749 10.1093/icvts/ivab086 PMC8923426

[8] Arakawa M, Akiyoshi K, Kitada Y, Miyagawa A, Okamura H. Comparison between Zone 2 and Zone 3 distal anastomoses for aortic arch replacement in terms of invasiveness. Gen Thorac Cardiovasc Surg. 2025;73:23–30. 38809376 10.1007/s11748-024-02045-7

[9] Lee KFL, Bhatia I, Chan TLD, Au WKT, Ho KLC. Proximalization of frozen elephant trunk procedure: Zone 0 or 1 versus zone 2 or 3 arch repair. Thorac Cardiovasc Surg. 2024;72:89–95. 36216330 10.1055/s-0042-1757631

[10] Yamamoto H, Kadohama T, Yamaura G, Tanaka F, Takagi D, Kiryu K, et al. Total arch repair with frozen elephant trunk using the zone 0 arch repair strategy for type A acute aortic dissection. J Thorac Cardiovasc Surg. 2020;159:36–45. 30902465 10.1016/j.jtcvs.2019.01.125

[11] Kato M, Kuratani T, Kaneko M, Kyo S, Ohnishi K. The results of total arch graft implantation with open stent-graft placement for type A aortic dissection. J Thorac Cardiovasc Surg. 2002;124:531–40. 12202870 10.1067/mtc.2002.124388

[12] Katayama K, Uchida N, Katayama A, Takahashi S, Takasaki T, Kurosaki T, et al. Multiple factors predict the risk of spinal cord injury after the frozen elephant trunk technique for extended thoracic aortic disease. Eur J Cardiothorac Surg. 2015;47:616–20. 24944331 10.1093/ejcts/ezu243

[13] Preventza O, Liao JL, Olive JK, Simpson K, Critsinelis AC, Price MD, et al. Neurologic complications after the frozen elephant trunk procedure: A meta-analysis of more than 3000 patients. J Thorac Cardiovasc Surg. 2020;160:20–e334. 31757456 10.1016/j.jtcvs.2019.10.031

[14] Takagaki M, Midorikawa H, Yamaguchi H, Nakamura H, Kadowaki T, Ueno Y, et al. Rapidly progressed distal arch aneurysm with distal open stent graft-induced new entry caused by “spring-back” force. Ann Vasc Dis. 2020;13:343–6. 33384744 10.3400/avd.cr.20-00075 PMC7751074

[15] Furutachi A, Osaki J, Koga K, Kamohara K. Late outcomes of the frozen elephant trunk technique for acute and chronic aortic dissection: the angle change of the FROZENIX by “spring-back” force. Gen Thorac Cardiovasc Surg. 2023;71:216–24. 35978158 10.1007/s11748-022-01863-x

[16] Gottardi R, Voetsch A, Krombholz-Reindl P, Winkler A, Steindl J, Dinges C, et al. Comparison of the conventional frozen elephant trunk implantation technique with a modified implantation technique in Zone 1. Eur J Cardiothorac Surg. 2020;57:669–75. 31504378 10.1093/ejcts/ezz234

[17] Okamura H, Kitada Y, Miyagawa A, Arakawa M, Adachi H. Clinical outcomes of a fenestrated frozen elephant trunk technique for acute type A aortic dissection. Eur J Cardiothorac Surg. 2021;59:765–72. 33284961 10.1093/ejcts/ezaa411

[18] Rockwood K, Song X, MacKnight C, Bergman H, Hogan DB, McDowell I, et al. A global clinical measure of fitness and frailty in elderly people. CMAJ. 2005;173:489–95. 16129869 10.1503/cmaj.050051 PMC1188185

[19] Sievers HH, Rylski B, Czerny M, Baier ALM, Kreibich M, Siepe M, et al. Aortic dissection reconsidered: type, entry site, malperfusion classification adding clarity and enabling outcome prediction. Interact Cardiovasc Thorac Surg. 2020;30:451–7. 31755925 10.1093/icvts/ivz281

[20] Kanda Y. Investigation of the freely available easy-to-use software “EZR” for medical statistics. Bone Marrow Transplant. 2013;48:452–8. 23208313 10.1038/bmt.2012.244 PMC3590441

[21] Okita Y, Kumamaru H, Motomura N, Miyata H, Takamoto S. Current status of open surgery for acute type A aortic dissection in Japan. J Thorac Cardiovasc Surg. 2022;164:785–e941. 33334600 10.1016/j.jtcvs.2020.09.147

[22] Ogino H, Okita Y, Uchida N, Kato M, Miyamoto S, Matsuda H, et al. Comparative study of Japanese frozen elephant trunk device for open aortic arch repairs. J Thorac Cardiovasc Surg. 2022;164:1681-92.e2. 33965229 10.1016/j.jtcvs.2021.03.079

[23] Roselli EE, Tong MZ, Bakaeen FG. Frozen elephant trunk for DeBakey type 1 dissection: the Cleveland Clinic technique. Ann Cardiothorac Surg. 2016;5:251–5. 27386416 10.21037/acs.2016.05.03 PMC4893536

[24] Hashizume K, Matsuoka T, Mori M, Takaki H, Koizumi K, Kaneyama H, et al. Total arch replacement with extended branched stented anastomosis frozen elephant trunk repair for type A dissection improves operative outcome. JTCVS Tech. 2022;17:1–9. 36820356 10.1016/j.xjtc.2022.10.011 PMC9938375

[25] Orozco-Sevilla V, Coselli JS. Management of the left subclavian artery during aortic arch replacement using a frozen elephant trunk approach: a review. Cardiovasc Diagn Ther. 2023;13:736–42. 37675092 10.21037/cdt-22-248 PMC10478019

[26] Bakalarz M, Rożniecki JJ, Stasiołek M. Clinical characteristics of patients with vertebral artery hypoplasia. Int J Environ Res Public Health. 2022;19:9317. 35954673 10.3390/ijerph19159317 PMC9368006

[27] Marshall RJ, Mantini EL. Dynamics of the collateral circulation in patients with subclavian steal. Circulation. 1965;31:249–54. 14261742 10.1161/01.cir.31.2.249

[28] Tsagakis K, Pacini D, Grabenwoger M, Borger MA, Goebel N, Hemmer W, et al. Results of frozen elephant trunk from the international E-vita Open registry. Ann Cardiothorac Surg. 2020;9:178–88. 32551250 10.21037/acs-2020-fet-25 PMC7298229

[29] Wada T, Yamamoto H, Takagi D, Kadohama T, Yamaura G, Kiryu K, et al. Aortic remodeling, reintervention, and survival after zone 0 arch repair with frozen elephant trunks for acute type A aortic dissection: midterm results. JTCVS Tech. 2022;14:29–38. 35967231 10.1016/j.xjtc.2022.05.013 PMC9366877

[30] Murana G, Gliozzi G, Di Marco L, Campanini F, Snaidero S, Nocera C, et al. Frozen elephant trunk technique using hybrid grafts: 15-year outcomes from a single-centre experience. Eur J Cardiothorac Surg. 2024;65:ezad364. 37930039 10.1093/ejcts/ezad364 PMC10859176

[31] Kreibich M, Berger T, Rylski B, Chen Z, Beyersdorf F, Siepe M, et al. Aortic reinterventions after the frozen elephant trunk procedure. J Thorac Cardiovasc Surg. 2020;159:392-9.e1. 30928219 10.1016/j.jtcvs.2019.02.069

[32] Haensig M, Schmidt A, Staab H, Steiner S, Scheinert D, Branzan D. Endovascular repair of the thoracic or thoracoabdominal aorta following the frozen elephant trunk procedure. Ann Thorac Surg. 2020;109:695–701. 31470013 10.1016/j.athoracsur.2019.07.011

[33] Woo W, Hong S, Kim TH, Baek MY, Song SW. Delayed pulmonary artery rupture after using BioGlue in cardiac surgery. Korean J Thorac Cardiovasc Surg. 2017;50:474–6. 29234619 10.5090/kjtcs.2017.50.6.474 PMC5716655

[34] Ngaage DL, Edwards WD, Bell MR, Sundt TM. A cautionary note regarding long-term sequelae of biologic glue. J Thorac Cardiovasc Surg. 2005;129:937–8. 15821668 10.1016/j.jtcvs.2004.07.046

[35] Ma WG, Ziganshin BA, Guo CF, Zafar MA, Sieller RS, Tranquilli M, et al. Does BioGlue contribute to anastomotic pseudoaneurysm after thoracic aortic surgery? J Thorac Dis. 2017;9:2491–7. 28932555 10.21037/jtd.2017.06.120 PMC5594166

[36] Malaisrie SC, Szeto WY, Halas M, Girardi LN, Coselli JS et al. Sundt TM 3rd,. 2021 The American Association for Thoracic Surgery expert consensus document: Surgical treatment of acute type A aortic dissection. J Thorac Cardiovasc Surg. 2021;162:735 – 58.e2. 10.1016/j.jtcvs.2021.04.053 34112502

[37] Kreibich M, Bunte D, Berger T, Vötsch A, Rylski B, Krombholz-Reindl P, et al. Distal stent graft-induced new entries after the frozen elephant trunk procedure. Ann Thorac Surg. 2020;110:1271–9. 32194032 10.1016/j.athoracsur.2020.02.017

[38] Osswald A, Schucht R, Schlosser T, Jánosi RA, Thielmann M, Weymann A, et al. Changes of stent-graft orientation after frozen elephant trunk treatment in aortic dissection. Eur J Cardiothorac Surg. 2021;61:142–9. 34329387 10.1093/ejcts/ezab297

[39] Takagi D, Yamamoto H, Kadohama T, Kiryu K, Wada T, Igarashi I. Optimal stent length and distal positioning of frozen elephant trunks deployed from the aortic zone 0 for type A acute aortic dissection. J Thorac Cardiovasc Surg. 2024;167:15–e252. 35422323 10.1016/j.jtcvs.2022.03.007

[40] Okita Y. Frozen elephant trunk with Frozenix prosthesis. Ann Cardiothorac Surg. 2020;9:152–63. 32551247 10.21037/acs.2020.03.13 PMC7298232

[41] Takei Y, Miyazaki S, Suzuki K, Saito S, Oogaki H, Muraoka Y, et al. Hemodynamic predictors of negative false lumen remodeling after frozen elephant trunk for acute aortic dissection. Gen Thorac Cardiovasc Surg. 2024;72:376–86. 37948001 10.1007/s11748-023-01984-x PMC11127806

