# Supplementary material for: Zone 1 total arch replacement with frozen elephant trunk for type A dissection: early-to-mid-term outcomes
Source: J Cardiothorac Surg. 2026 Jun 3;21:564. doi: 10.1186/s13019-026-04324-1 (PMC13474498; doi:10.1186/s13019-026-04324-1)
Supplement: Supplementary file 1 [file 13019_2026_4324_MOESM2_ESM.docx]

**Additional file 2. Examples of computed tomography measurements**

(A) Measurement of the FET and spring-back angles. The FET angle was defined as the topmost angle of a triangle formed by the proximal edge, distal edge, and top of the FET on the lesser curvature side. The spring-back angle was defined as the angle formed between the native aorta and distal FET. Coaxial measurement of the maximum short-axis diameter in (B) zone 4 and (C) zone 5. FET, frozen elephant trunk.
