# Supplementary material for: Zone 1 total arch replacement with frozen elephant trunk for type A dissection: early-to-mid-term outcomes
Source: J Cardiothorac Surg. 2026 Jun 3;21:564. doi: 10.1186/s13019-026-04324-1 (PMC13474498; doi:10.1186/s13019-026-04324-1)
Supplement: Supplementary file 2 [file 13019_2026_4324_MOESM3_ESM.docx]

**Additional file 3. Kaplan–Meier curves showing overall survival**

(a) Overall survival of the entire cohort, with one-, three-, and five-year survival rates of 87.3%, 85.6%, and 85.6%, respectively. (b) Overall survival stratified by the presence or absence of MPS. Patients without MPS had one-, three-, and five-year survival rates of 91.1%, 89.2%, and 89.2%, respectively, whereas those with MPS (M1 and/or M2) had markedly lower survival rates (63.6% at one, three, and five years). CI, confidence interval; MPS, malperfusion syndrome; M1, coronary malperfusion; M2, supra-aortic malperfusion.
