# Supplementary material for: Zone 1 total arch replacement with frozen elephant trunk for type A dissection: early-to-mid-term outcomes
Source: J Cardiothorac Surg. 2026 Jun 3;21:564. doi: 10.1186/s13019-026-04324-1 (PMC13474498; doi:10.1186/s13019-026-04324-1)
Supplement: Supplementary file 3 [file 13019_2026_4324_MOESM4_ESM.docx]

**Additional file 4. Cox proportional hazards analysis of overall survival**

| **Variable** | **Hazard ratio** | **95% CI** | **p value** |
| --- | --- | --- | --- |
| Presence of M1 and/or M2 | 4.86 | 1.34–17.61 | 0.016 |
| Age (per one-year increase) | 0.99 | 0.94–1.05 | 0.77 |
| Male sex | 0.43 | 0.10–1.86 | 0.26 |

CI, Confidence interval; M1, coronary malperfusion; M2, supra-aortic malperfusion.
