# Supplementary material for: Zone 1 total arch replacement with frozen elephant trunk for type A dissection: early-to-mid-term outcomes
Source: J Cardiothorac Surg. 2026 Jun 3;21:564. doi: 10.1186/s13019-026-04324-1 (PMC13474498; doi:10.1186/s13019-026-04324-1)
Supplement: Supplementary file 4 [file 13019_2026_4324_MOESM5_ESM.docx]

**Additional file 5**. **Details of spinal cord injury cases**

| **Case** | **Age / Sex** | **Diagnosis** | **Lower body circulatory arrest time (min)** | **FET distal tip location** | **SCI type** |
| --- | --- | --- | --- | --- | --- |
| 1 | 55 M | ATAAD (DeBakey I) | 50 | Th5/6 | Paraparesis |
| 2 | 38 M | ATAAD (DeBakey I) | 107 | Th8 | Paraplegia |
| 3 | 74 M | ATAAD (DeBakey I) | 78 | Th5/6 | Paraplegia |
| 4^†^ | 80 M | ATAAD (DeBakey I) | 34 | Th6 | Paraplegia (preoperative) |

†Case 4 had preoperative paraplegia due to a thrombosed intramural hematoma and is distinct from the three postoperative SCI cases. ATAAD, acute type A aortic dissection; FET, frozen elephant trunk; M, male; SCI, spinal cord injury; Th, thoracic vertebra.

**Case descriptions:**

**Case 1 (Paraparesis):** A 55-year-old man who developed lower limb weakness postoperatively. Lower body circulatory arrest time was 50 minutes, and the FET distal tip was positioned at Th5/6. The specific cause remains unclear, as neither the circulatory arrest time nor the FET tip position was clearly in the high-risk range.

**Case 2 (Paraplegia):** A 38-year-old man with inadvertent deep FET deployment (distal tip at Th8). The non-stented graft segment developed kinking and stenosis, resulting in reduced distal perfusion pressure and spinal cord ischemia. Supplementary TEVAR was performed to address the kinking, but paraplegia persisted. This case was attributed to a technical complication.

**Case 3 (Paraplegia):** A 74-year-old man with a dorsal false lumen from which multiple intercostal arteries originated. Despite successful false lumen thrombosis confirmed on postoperative CT, thrombosis of the dorsal false lumen likely interrupted perfusion to multiple intercostal arteries, leading to spinal cord ischemia.

**Case 4 (Preoperative paraplegia):** An 80-year-old man who presented with preoperative paraplegia due to a thrombosed intramural hematoma compressing the spinal cord. This case is distinct from the three cases of postoperative SCI.
