# Supplementary material for: Zone 1 total arch replacement with frozen elephant trunk for type A dissection: early-to-mid-term outcomes
Source: J Cardiothorac Surg. 2026 Jun 3;21:564. doi: 10.1186/s13019-026-04324-1 (PMC13474498; doi:10.1186/s13019-026-04324-1)
Supplement: Supplementary file 5 [file 13019_2026_4324_MOESM6_ESM.docx]

**Additional file 6. Postoperative computed tomography showing left subclavian artery bypass occlusion**

(a) Late occlusion without kinking or graft narrowing observed 7.5 years postoperatively. Occlusion observed (b) six months postoperatively and (c,d) two years postoperatively, with graft kinking at the proximal portion and narrowing at the intercostal level. Arrows indicate the sites of graft kinking, and circles indicate narrowing caused by intercostal compression.
